# The Role of Serum Erythropoietin (EPO) and Vascular Endothelial Growth Factor (VEGF) in Pulse Wave Velocity (PWV) Among Hypertensive Patients: A Cross-Sectional Study

**DOI:** 10.7759/cureus.62416

**Published:** 2024-06-15

**Authors:** Sumangala M Patil, Jyoti P Khodnapur, Kusal K Das, Amrit Podder

**Affiliations:** 1 Physiology, Shri B M Patil Medical College, Hospital and Research Centre, Vijayapur, IND; 2 Physiology, Teerthanker Mahaveer Medical College and Research Centre, Moradabad, IND

**Keywords:** arterial stiffness, oxygen sensing proteins, endothelial dysfunction, oxidative stress, vascular endothelial growth factor (vegf), erythropoietin (epo), pulse wave velocity (pwv), malondialdehyde (mda), nitric oxide (no), hypertension

## Abstract

Background and objective

While hypertension (HTN) is a major health-related threat globally, it is often an under-reported clinical condition as most of the stage I hypertensive patients do not present with any symptoms. The relationship between endogenous oxygen-sensing protein [erythropoietin (EPO) and vascular endothelial growth factor (VEGF)] levels and vascular stress in hypertensive patients is not fully understood as the mechanistic pathway by which these oxygen-sensing proteins alter the vascular physiology and cause hypertension is still a matter of debate. In light of this, we explored the role of these two proteins in the development of vascular stress including increased pulse wave velocity (PWV). We aimed to examine the correlation between oxygen-sensing proteins and vascular stress markers including PWV in hypertensive patients.

Materials and methods

We conducted a cross-sectional study involving age-matched participants classified into three groups (group 1: normotensive persons, n=36; group 2: stage I hypertensive patients, n=36; and group 3, stage II hypertensive patients, n=36). Adiposity-related parameters such as waist circumference (WC), hip circumference (HC), BMI, and waist-hip ratio (WHR) were measured. BP was recorded manually in resting posture by using a sphygmomanometer. PWV, which predicts the progression of BP and the development of HTN, was recorded using a periscope, which works based on the oscillometric method. Vascular stress-induced oxidative stress parameters [serum malondialdehyde (MDA) and serum nitric oxide (NO)] were also estimated by using a UV spectrophotometer. Quantitative estimations of oxygen-sensing proteins (serum EPO and serum VEGF) were done by using the ELISA kit method. The results were expressed as mean ± standard deviation (SD). The correlation between the variables was done using Spearman’s correlation. A p-value <0.05 was considered statistically significant.

Results

Adiposity indices and vascular stiffness parameters were found to be significantly (p <0.05) increased in group 2 and group 3 compared to group 1. The levels of serum MDA were found to be significantly (p<0.05) increased in group 2 and group 3 than group 1, whereas the levels of serum NO were significantly (p<0.05) decreased in group 3 and group 2 than group 1. A significant (p<0.05) positive correlation was observed between the PWV and EPO (r=0.492) while a significant (p<0.05) negative correlation was observed between PWV and VEGF (r=-0.406) among the study population.

Conclusion

The results are indicative of the influence of vascular stress in stage I and II hypertensive patients. Furthermore, the relationship between oxygen-sensing proteins and vascular stress in hypertensive patients has also been established.

## Introduction

Hypertension (HTN) is the most common risk factor for death due to stroke and cardiovascular diseases (CVD). It is one of the major rising causes of secondary illnesses that lead to morbidity and mortality [1]. Increased vascular stiffness is a strong predictor of the potential development of HTN as well as an early marker of systemic atherosclerosis [2]. Several studies have shown that hypertensive patients have lower levels of serum nitric oxide (NO) and higher oxidative stress, which alter the vascular architecture [3]. It is well-known that erythropoietin (EPO) has a significant role in the physiological maintenance of the cardiovascular system, but the relationship between serum EPO levels and vascular stress has been a matter of debate for a long time [4]. Studies also show that vascular endothelial growth factor (VEGF) might play an important role in the development of vascular abnormalities [5].

Emerging experimental data and data from various clinical studies indicate that EPO and VEGF have a significant role in the cardiovascular system; e.g., VEGF signaling pathway inhibition induces hypertension, EPO therapy results in increased BP, and EPO-induced hematocrit values and erythrocyte mass alter the integrity of vascular smooth muscles, leading to the deregulation of endothelial vasodilatory factors [4,5,6]. The relationship between EPO and VEGF levels and vascular stress has not been fully explored. Hence, in this study, we aimed to assess the association of oxygen-sensing proteins (EPO and VEGF) with vascular stress parameters including pulse wave velocity (PWV) in hypertensive patients.

## Materials and methods

Study design

This was a cross-sectional study conducted in the Laboratory of Vascular Physiology and Medicine, Department of Physiology, Shri B M Patil Medical College Hospital and Research Centre, BLDE (Deemed to be University), Vijayapura, Karnataka, India from May 1, 2021, to October 31, 2022. The participants were selected through a comprehensive sampling method. We included 108 participants of both genders (54 males and 54 females) divided into three groups based on the latest classification of hypertension by the American Heart Association (AHA) [1,2]: group 1 (normotensives, n=36), group 2 (stage I hypertensives, n=36), and group 3 (stage II hypertensives, n=36). The age of the cohort ranged from 35 to 50 years.

Ethical consideration

Ethical clearance was obtained from the Institutional Ethics Committee of BLDE (Deemed to be University) (IEC/No-09/2021, dated 22/01/2021). Voluntary written informed consent was taken from all the participants.

Inclusion and exclusion criteria

Participants in the age group of 35 to 50 years who were willing to provide voluntary informed written consent were included in our study. Chronic smokers, alcoholics, diabetics, and patients with thyroid diseases were excluded. Patients taking antihypertensive treatments were also excluded as the antihypertensive drugs may modulate the oxygen-sensing mechanism of the vessels.

Procedure and assessment

All the anthropometric parameters, vascular stiffness parameters, and blood pressure phenotypes were recorded with patients in the supine posture after resting for 10 minutes between 6 AM and 7 AM at room temperature, following which the overnight fasting blood samples were also collected from each participant between 7 AM and 8 AM. The blood samples were assessed for parameters such as oxidative stress and oxygen-sensing protein levels.

Sample size

With the anticipated correlation coefficient between EPO and PWV - 0.555 at a 95% confidence level and 90 power in the study, the sample size was determined to be 36 per group [4]. The calculation of the total sample size was as follows: 36+36+36=108

Assessed parameters

I. Anthropometry [2]

A. Height: Height was measured using a device (BIOCONTM) mounted on the wall and expressed in cm. B. Weight: Weight was measured using a weighing machine and expressed in kg. C. BMI: BMI was calculated manually as follows: weight in kg divided by height in meters squared (m^2^) and expressed as kg/m^2^. D. Waist circumference (WC) in cm. E. Hip circumference (HC) in cms. F. Waist-hip ratio (WHR).

II. Blood Pressure Phenotypes

Systolic blood pressure (SBP, mmHg) and diastolic blood pressure (DBP, mmHg) were recorded using a mercury sphygmomanometer. All the parameters were recorded three times for each of the participants and the average value was considered. Pulse pressure (PP) was defined as the difference between SBP and DBP in mmHg and the mean arterial pressure (MAP) was calculated [7].

III. Vascular Stress

Vascular Stiffness

Pulse wave velocity (PWV): PWV was measured by using a periscope (Genesis Medical Systems, Hyderabad, India), an automated instrument that works on oscillometric method, and was reported as right brachial-ankle PWV (PWVb-a Right), left brachial-ankle PWV (PWVb-a Left), and carotid-femoral PWV (PWVc-f) [8]. All recordings were done in the supine position and operational bias was avoided as this device is fully automated.

Oxidative Stress

Serum MDA was measured by the TBARS method using a UV spectrophotometer at 535 nm. Serum NO levels were estimated by using a spectrophotometer (Shimadzu UV 800) at 535 nm [9].

Oxygen-sensing proteins: Quantitative estimation of serum VEGF and serum EPO was done by enzyme-linked immunosorbent assay (ELISA) with a kit method [10].

Statistical analysis

The obtained data were entered into an MS Excel sheet. Statistical analysis was performed using SPSS Statistics version 20 (IBM Corp., Armonk, NY). Data were presented as mean ± standard deviation (SD). Categorical variables were compared by using the Chi-square test. Differences between groups of continuous variables were compared using the Kruskal-Wallis test. Spearman’s correlation was performed between the variables of vascular stress parameters and oxygen-sensing proteins and also between blood pressure phenotypes and oxygen-sensing proteins. A p-value <0.05 was considered statistically significant. All the statistical tests were two-tailed.

## Results

Descriptive analysis of parameters

Table 1 shows the comparison between parameters of anthropometry and BP phenotypes (SBP, DBP, PP, MAP) of the three groups. The was a significant (p<0.05) increase in the adiposity indices (parameters of anthropometry) in group 2 than group 1 and in group 3 than group 2. The significant (p<0.05) increase in BP in group 2 than in group 1 and group 3 than group 2 make it evident that the participants of our study were properly selected. The comparison of the vascular stress parameters and oxygen-sensing proteins between all three groups is depicted in Table 2. It shows that the vascular stiffness (PWV) was significantly (p<0.05) increased in group 2 than in group 1 and in group 3 than group 2. It also shows that the oxidative stress parameter (serum MDA) was significantly (p<0.05) increased and the serum NO significantly (p<0.05) decreased in group 2 and group 3 than in group 1. Table 2 also depicts the significantly increased level of serum EPO and decreased level of serum VEGF in group 2 than in group 1 and in group 3 than group 2.

**Table 1:** Physical anthropometry and physiological parameters in all three groups P-value <0.05 is considered statistically significant SD: standard deviation; HTN: hypertension; BMI: body mass index; WC: waist circumference; HC: hip circumference; WHR: waist-hip ratio; SBP: systolic blood pressure; DBP: diastolic blood pressure; PP: pulse pressure; MAP: mean arterial pressure

Parameters	Normotensives (n=36), mean ± SD	Stage I HTN (n=36), mean ± SD	Stage II HTN (n=36), mean ± SD	P-value
BMI (Kg/m^2^)	23.06 ± 1.31	25.81 ± 3.87	27.9 ± 3.21	<0.0001
WC (cm)	84.17 ± 1.59	92.51 ± 9.08	97.5 ± 5.68	<0.0001
HC (cm)	85.97 ± 2.91	94.08 ± 9.65	97.8 ± 6.59	<0.0001
WHR	0.97 ± 0.03	0.98 ± 0.05	0.99 ± 0.04	<0.0001
SBP (mmHg)	115.1 ± 3.39	134.2 ± 2.762	148.1 ± 8.342	<0.0001
DBP (mmHg)	73.28 ± 4.76	83.17 ± 3.621	90.33 ± 6.076	<0.0001
PP (mmHg)	41.83 ± 5.05	51.00 ± 4.623	57.72 ± 10.33	<0.0001
MAP (mmHg)	87.22 ± 3.65	100.2 ± 2.684	109.7 ± 4.911	<0.0001

**Table 2:** Vascular stress parameters and oxygen-sensing proteins among all three groups P-value <0.05 is considered statistically significant SD: standard deviation; HTN: hypertension; PWVb-a Right: pulse wave velocity brachial-ankle right side; PWVb-a Left: pulse wave velocity brachial-ankle left side; PWVc-f: pulse wave velocity carotid-femoral; MDA: malondialdehyde; NO: nitric oxide; EPO: erythropoietin; VEGF: vascular endothelial growth factor

Parameters	Normotensives (n=36), mean ± SD	Stage I HTN (n=36), mean ± SD	Stage II HTN (n=36), mean ± SD	P-value
PWV_b-a_ Right (cm/sec)	1170.7 ± 234	1345 ± 152	1544 ± 246	<0.0001
PWV_b-a_ Left (cm/sec)	1136.6 ± 107	1288 ± 162	1418 ± 373	<0.0001
PWV_c-f_ (cm/sec)	738.73 ± 98	874.2 ± 139	989.7 ± 175	<0.0001
Serum MDA (μmol/L)	1.014 ± 0.2	1.222 ± 0.4	2.083 ± 0.55	<0.0001
Serum NO (μmol/L)	8.532 ± 1.5	5.694 ± 1.4	3.694 ± 1.16	<0.0001
Serum EPO (pg/ml)	105.6 ± 25.3	132.6 ± 18.9	151.8 ± 22.1	<0.0001
Serum VEGF (pg/ml)	410.7 ± 44.1	380.1 ± 26.5	343.3 ± 39.2	<0.0001

Correlation between parameters

Table 3 depicts the correlation of different pulse wave velocities (PWVb-a Right, PWVb-a Left, PWVc-f) with oxygen-sensing proteins (EPO, VEGF) among all study participants. Our results indicate a significant (p<0.05) positive correlation between different pulse wave velocities with serum EPO while demonstrating a significant (p<0.05) negative correlation between different pulse wave velocities with serum VEGF. BP phenotypes were found to be significantly (p<0.05) increased with increased PWV.

**Table 3:** Correlation between vascular stiffness with blood pressure phenotype and oxygen-sensing proteins P-value <0.05 is considered statistically significant (-): negatively correlated; r: correlation coefficient; PWVb-a Right: pulse wave velocity brachial-ankle right side; PWVb-a Left: pulse wave velocity brachial-ankle left side; PWVc-f: pulse wave velocity carotid-femoral; SBP: systolic blood pressure; DBP: diastolic blood pressure; PP: pulse pressure; MAP: mean arterial pressure; EPO: erythropoietin; VEGF: vascular endothelial growth factor

Parameters	PWV_b-a_ Right (cm/sec)	PWV_b-a_ Left (cm/sec)	PWV_c-f_ (cm/sec)
SBP (mmHg)	r=0.528 (n=108, p<0.0001)	r=0.447 (n=108, p<0.0001)	r=0.568 (n=108, p<0.0001)
DBP (mmHg)	r=0.447 (n=108, p<0.0001)	r=0.424 (n=108, p<0.0001)	r=0.478 (n=108, p<0.0001)
PP (mmHg)	r=0.456 (n=108, p<0.0001)	r=0.357 (n=108, p<0.0001)	r=0.486 (n=108, p<0.0001)
MAP (mmHg)	r=0.510 (n=108, p<0.0001)	r=0.432 (n=108, p<0.0001)	r=0.540 (n=108, p<0.0001)
EPO (pg/ml)	r=0.492 (n=108, p<0.0001)	r=0.327 (n=108, p<0.0001)	r=0.296 (n=108, p<0.0001)
VEGF (pg/ml)	r=-0.406 (n=108, p<0.0001)	r=-0.256 (n=108, p<0.0001)	r=-0.405 (n=108, p<0.0001)

## Discussion

Several mechanisms might play a role in increased adiposity indices as the blood pressure increases, such as increased vascular age, atherosclerosis, and altered vascular architecture [11].

Vascular stress parameters

PWVb-a Right, PWVb-a Left, and PWVc-f of stage II and stage I HTN group were significantly higher compared to the normotensive group, which indicates the deviation of vascular integrity from normal in both HTN groups. Higher vascular stiffness in stage II HTN indicates pathophysiological changes in the vascular system. Melo E Silva et al. (2021) established an association of body composition with arterial stiffness and a positive correlation exists between obesity and arterial stiffness, which may lead to cardiovascular risks such as HTN. These findings align with our results [12]. Vascular stiffness is also an indicator of early CVD, dementia, and possibly fatal outcomes. PWVb-a Right, PWVb-a Left, and PWVc-f of stage II patients were found to be higher than the stage I HTN group. Furthermore, the stage I HTN group also showed significantly higher vascular stiffness parameters than the normotensive group. The results indicate impairment of vascular integrity compared to controls. Increased PWV also indicates stiffness of conduit arteries, which is a risk factor for CVD [13].

PWV generation occurs due to the contraction of the heart. Later, pulse waves travel through the vascular wall at a particular speed, which is referred to as PWV. An increase in vascular stiffness results in arterial compliances, and leads to an increase in PWV. Loss of integrity in vascular walls causes loss of elasticity of the arteries and makes arteries stiff. More stiffer vascular wall will lead to higher PWV. This pathophysiology changes cardiac functioning and leads to overall de-arrangement of the cardiovascular system [14]. Furthermore, these de-arrangements will lead to HTN. Our results indicate the altered pathophysiology of the cardiovascular system in hypertensive individuals. The decreased elasticity of the vessels makes the blood vessels more vulnerable to cardiovascular and cerebrovascular risk factors.

Oxidative and nitrosative stress parameters in stage I and stage II hypertensive patients were found to be remarkably altered. Increased MDA in both stage I and stage II HTN indicate altered vascular pathophysiology. Excessive MDA in stage I and stage II HTN in our study may be due to the generation of more reactive oxygen species (ROS), which plays a key role in HTN pathology by modulating the vasomotor system and developing vasoconstriction through angiotensin II. Lower NO levels in stage I and stage II HTN patients indicate lesser bioavailability of NO, which is a potent vasodilator and extremely dependent on the redox signaling system. Increased levels of ROS in our study may have induced vascular remodeling via oxidative damage. Hence, both MDA and NO results in our study confirm the alteration of arterial smooth muscle cells and endothelial cells. The NO-related results are also to be considered as a degree of HTN, and, possibly, the antioxidant status might have changed simultaneously during HTN [15].

Parameters of oxygen-sensing proteins

Increased levels of serum EPO in both stage I and stage II hypertensive patients indicate the loss of vascular integrity due to HTN. Although different types of explanations were given for the rise of BP or HTN and increased level of EPO, probably due to reduced oxygen supply to the tissue in vasoconstriction-induced HTN, the role of altered angiogenesis may not be ruled out for a positive correlation between BP and serum EPO concentration, which may be due to decreased angiogenesis. One of the reasons for elevated blood pressure by EPO is its vasoconstrictor effects on small resistance vessels and further negative impact on acetylcholine-induced vasodilatory effects. Reports also suggest that serum EPO level has a positive correlation with vascular resistance, which may also lead to HTN. EPO-induced hematocrit values and erythrocyte mass may alter the integrity of vascular smooth muscles, leading to the deregulation of endothelial vasodilatory factors like NO.

Our results showed that an increased level of serum EPO may stimulate increased angiotensinogen activities, leading to increased production of angiotensin II and subsequently excessive sodium retention in the body and HTN [16]. Our results showing low NO probably support this observation. In our results, an increased level of serum EPO indicates excessive production of EPO from the kidney. Increased level of EPO exerts hypertensinogenic effects [17]. It has been observed that treating chronic kidney disease (CKD) patients with EPO may lead to severe arterial HTN. This could be attributed to EPO-induced increased blood viscosity and decreased hypoxic vasodilatation [18]. Furthermore, it has also been noted that EPO increases calcium ion influx via opening L-type calcium channels in vascular smooth muscle cells and causes vasoconstriction [19].

The results showed serum VEGF decreases in both stage I and stage II HTN. VEGF protein synthesis depends on the hypoxia signaling pathway that regulates arterial smooth muscle pathophysiology. Hence, an alteration in VEGF indicates cardiovascular and cerebrovascular diseases. In our study, lower levels of VEGF probably influenced reduced vasculogenesis and remodeled vascular architecture in stage I and stage II HTN [20]. The report also found that VEGF inhibition leads to HTN as decreased VEGF also reduces NO synthesis and microvascular abnormalities, and increases vascular resistance, leading to the development of HTN [19]. Our results showing lower levels of NO and VEGF support these findings. Another possible reason for VEGF signaling NO synthesis is VEGF receptor (VEGFR) [20]. Decreased VEGF leads to NO signal pathways reducing NO secretion pathway, causing hypertension, although it is still debatable [21]. It has been observed that anti-VEGFR-2 antibody treatment in mice caused hypertension due to reduced NOS3 and NOS1 expression in the kidney [22]. Although the exact reason pertaining to HTN and VEGF is not clearly defined, serum VEGF needs to be considered as one of the important markers for progressive HTN, especially the transformation of HTN stage I and stage II [23].

Limitations of the study

This study has a few limitations. We did not assay VEGFR in serum; hence, we cannot explain the role of VEGFR-induced HTN. Also, did not assess the level of serum eNOS (endothelial nitric oxide synthase), which is also a limitation besides the relatively smaller sample size in our study.

## Conclusions

Our findings point to altered anthropometric parameters as risk factors for vascular stiffening and future adverse cardiovascular diseases such as HTN. The results suggest a relationship between oxygen-sensing proteins (EPO and VEGF) and PWV in hypertensive patients. Hence, PWV and molecular parameters may be considered for the screening of patients suspected to be at risk of cardiovascular diseases in the future.
